# Necrostatin-1 Alleviates Diffuse Pulmonary Haemorrhage by Preventing the Release of NETs via Inhibiting NE/GSDMD Activation in Murine Lupus

**DOI:** 10.1155/2023/4743975

**Published:** 2023-03-01

**Authors:** Xinai Han, Xiaoming Zhang, Rui Song, Shiqi Li, Shujing Zou, Quanguang Tan, Tianyang Liu, Shugeng Luo, Zhe Wu, Hongyu Jie, Jinhong Wang

**Affiliations:** ^1^Department of Rheumatology and Immunology, The Third Affiliated Hospital, Southern Medical University, Guangzhou, 510630 Guangdong, China; ^2^Provincial Key Laboratory of Bone and Joint Degeneration Diseases, The Third Affiliated Hospital, Southern Medical University, Guangzhou, 510630 Guangdong, China; ^3^Department of Respiration, The Third Affiliated Hospital, Southern Medical University, Guangzhou 510630, China

## Abstract

Diffuse alveolar haemorrhage (DAH) is a rapidly developing condition owing to a lack of effective treatment and resulting in a high mortality rate in systemic lupus erythematosus (SLE). Neutrophil extracellular traps (NETs) contain numerous antigens and proinflammatory substances that directly damage the vascular endothelium and aggravate vascular inflammation, which is considered an important pathogenic factor of DAH in SLE. Therefore, blocking the release of NETs from neutrophils is an important target for the treatment of DAH in SLE. In this study, we investigated whether the inhibition of neutrophils releasing NETs could relieve DAH in SLE. Necrostatin-1 (Nec-1), a small molecule, has been reported to inhibit the release of NETs by neutrophils. In vitro experiments revealed that Nec-1 inhibited alveolar epithelial cell damage by preventing the release of NETs. Furthermore, vivo studies showed that Nec-1 alleviated lupus pulmonary haemorrhage in mice by reducing lung pathology severity, body weight, and serum inflammatory cytokine levels. Mechanistically, Nec-1 prevented NET release by inhibiting neutrophil elastase (NE) activation and N-Gasdermin D (N-GSDMD) expression. Additionally, immunohistochemistry and immunofluorescence findings showed that Nec-1 decreased NE expression in the lung tissues of mice with lupus pulmonary haemorrhage. Thus, NETs released by neutrophils contributed to the pathogenesis of DAH in SLE, and Nec-1 showed protective effects by the inhibition of NET production via the reduction of NE activation and N-GSDMD expression.

## 1. Introduction

Diffuse alveolar haemorrhage (DAH) is a serious complication of systemic lupus erythematosus (SLE) [1, 2], with approximately 3% of DAH-associated deaths reported in patients with SLE [3]. However, effective treatment for DAH remains elusive. Previous studies have reported that neutrophil extracellular traps (NETs) as an important source of autoantigens in lupus contribute to the pathogenesis of SLE [4–7]. NETs contain a large number of antigens and inflammatory substances that directly damage the vascular endothelium and increase vascular permeability [8–10]. The NETs released by neutrophils have a major effect on pulmonary haemorrhage in lupus, owing to pulmonary capillary inflammation [11, 12]. Therefore, inhibiting the formation of NETs could be an important target in the treatment of pulmonary haemorrhage in SLE [13].

The formation of NETs is associated with multilevel signalling pathways. Currently, the targets reported in the literature that could inhibit the formation of NETs mainly include the following aspects: (1) Ca^2+^ channel inhibitors; (2) PAD4 inhibitors; (3) NADPH oxidase inhibitors; and (4) Transcription inhibitors, such as AKT, P38, ERK, and other inhibitors [14–16]. Despite the varied and numerous targets reported, no effective treatment methods are available [17]. Neutrophil elastase (NE) is involved in chromatin decondensation, which eventually leads to cell membrane rupture and cellular release of NETs [18, 19]. A recent study indicated that NE cleaved the poring protein Gasdermin-D (GSDMD) to form N-terminal-GSDMD (N-GSDMD), which promotes the perforation and rupture of the neutrophil membrane and subsequently causes the release of NETs [20]. Necrostatin-1 (Nec-1) is an inhibitor of programmed necrosis. A previous study reported that Nec-1 could inhibit NET formation [21]. We supposed that maybe Nec-1 inhibited the release of NETs through NE [22]. Nonetheless, it remains unclear whether Nec-1 reduces the production of NETs by inhibiting the activation of the NE/GSDMD pathway.

NETs are rich in serine proteases (such as NE) and matrix metalloproteinases (MMPs), which directly damage vascular endothelial cells and increase vascular permeability. This ultimately leads to disease progression and organ damage in lupus [5, 9, 23]. NET-bound tissue factors can activate platelets and coagulation factors, promote thrombosis, and lead to pulmonary haemorrhage in lupus [24, 25]. Moreover, the effect of Nec-1 in reducing the release of NETs has been indicated as a significant curative strategy in neutrophilic asthma [20]. In the study, we aim to explore the role of Nec-1 in treating DAH by reducing NET release and investigate the molecular mechanism underlying this phenomenon.

## 2. Results

### 2.1. NETs Damage Alveolar Epithelial Cells in a Dose-Dependent Manner

**Figure 1:**
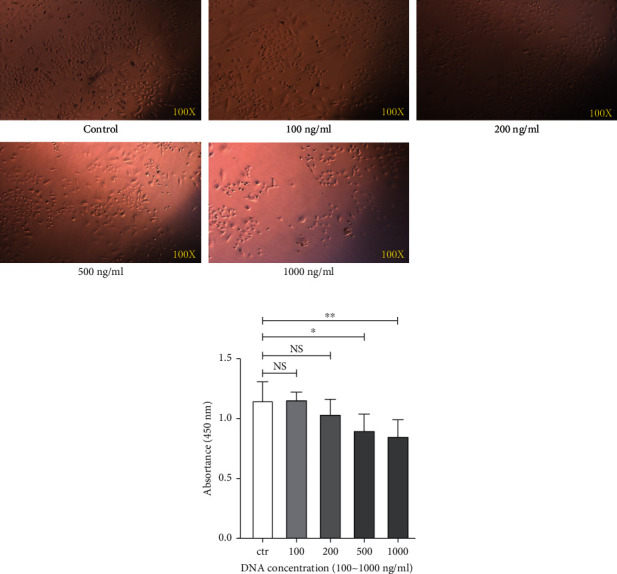
Neutrophil extracellular traps (NETs) damage alveolar epithelial cells in a dose-dependent manner. (a) A549 cells were cultured with different concentrations of NETs, and the morphological changes were observed under an optical fibre microscope. Representative images of the A549 cells are shown. (b) The proliferation of A549 cells was further detected using an enzyme-plate analyser. Values are expressed as mean ± standard error of the mean (SEM) of three independent experiments (^∗^*P* < 0.05 and ^∗∗^*P* < 0.01; NS: not significant).

Previous studies report that NETs could damage the vascular endothelium and increase vascular permeability [20]. In this study, we used A549 cells (human lung adenocarcinoma cells) for the assessment of cellular damage by NETs. Different concentrations of NETs (from 100 ng/mL to 1000 ng/mL) were treated with A549 cells for 24 h. Under an optical fibre microscope, A549 cells were observed to become rounder, less adherent, and less active in the high NET concentration groups (500 ng/mL and 1000 ng/mL) compared to the lower NET concentration and control groups (Figure 1(a)). The cell proliferation was further detected using an enzyme-plate analyser. The number of viable cells in the 500 ng/mL and 1000 ng/mL NET treatment groups were significantly reduced compared to the control and 100 ng/mL NET groups (*P* < 0.05) (Figure 1(b)). Thus, these results indicated that the proliferation of A549 cells was impaired in the NET concentrations above 500 ng/mL.

### 2.2. Nec-1 Significantly Inhibited NET Formation In Vitro

**Figure 2:**
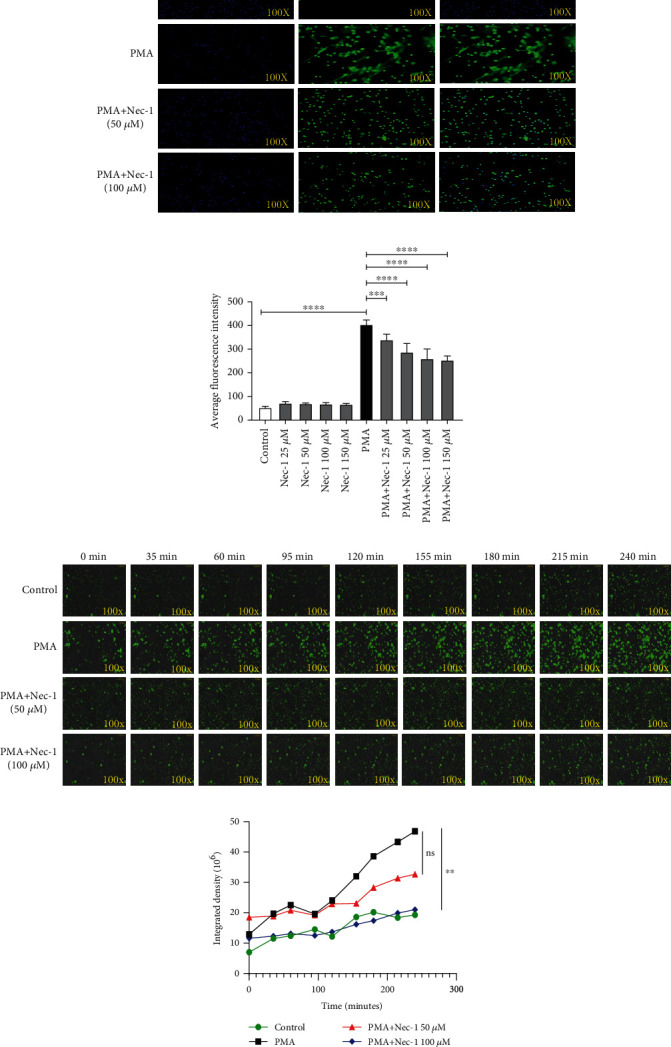
Nec-1 inhibited the formation of neutrophil extracellular traps (NETs) *in vitro*. (a) Neutrophils were cultured with phorbol 12-myristate 13-acetate (PMA) or PMA plus Nec-1 (50 *μ*M or 100 *μ*M). Representative immunofluorescence images of neutrophils stained using SYTOX Green (green) and Hoechst (blue) (200x). (b) Peripheral blood neutrophils were cultured with PMA or PMA plus different concentrations of Nec-1 (50 or 100 *μ*M). Extracellular DNA levels, which were determined via SYTOX Green staining, were detected using a microplate reader. (c) SYTOX Green-stained neutrophils stimulated with 25 nM PMA or with different concentrations of Nec-1 (50 or 100 *μ*M) were photographed for 4 h using an automatic live-cell imaging analysis system with the GFP green channel (measurement of non-transmembrane SYTOX Green). The green fibrin-like structures indicate NETs. Videos S1–S4 show the change of untreated cells and also of cells treated with PMA (25 nM), PMA+Nec-1 (50 *μ*M), and PMA+Nec-1 (100 *μ*M), respectively. (d) The mean fluorescence intensity of live-cell imaging pictures at different time points is presented and analysed (0-240 min). The mean fluorescence intensity of SYTOX Green at different time points was determined using ImageJ semiquantitative statistics. Values are expressed as mean ± standard error of the mean (SEM) of three independent experiments (^∗∗∗^*P* < 0.001 and ^∗∗∗∗^*P* < 0.0001).

Previous studies indicated that Nec-1 inhibited programmed cell necrosis by targeting RIPK1 kinase [6, 20, 26]. In this study, we found that Nec-1 could inhibit NET formation stimulating by phorbol 12-myristate 13-acetate (PMA) in vitro. The cells treated with PMA (25 nM) plus Nec-1 (50 *μ*M, 100 *μ*M, and 150 *μ*M) exhibited less SYTOX Green fluorescence compared with those stimulated by PMA alone (Figure 2(a)). Conversely, more cells were stained by Hoechst in the PMA plus Nec-1 group. The related quantitative results also suggested that cells treated with PMA (25 nM) plus Nec-1 (50 *μ*M, 100 *μ*M, or 150 *μ*M) had a lower fluorescence intensity of SYTOX Green than those stimulated by PMA alone (*P* < 0.001) (Figure 2(b)). Moreover, no significant difference in average fluorescence intensity between the control and Nec-1 (25 *μ*M, 50 *μ*M, 100 *μ*M, and 150 *μ*M) groups was observed.

To observe the morphological changes in PMA-stimulated neutrophils, a living cell imaging system was used to capture images of SYTOX Green-stained cells dynamically from 0 to 240 min (Figure 2(c) and Supplementary 1). The nuclei of neutrophils in the PMA group were condensed, and the cells appeared to rapidly swell, followed by nuclear depolymerization, and disintegrated, and finally, the cell membranes ruptured. In the process of nuclear expansion and disintegration, it was observed that the nonpermeable nucleic acid dye SYTOX Green could enter the cell before the cell membrane ruptured. Moreover, with the increase in the degree of nuclear depolymerization and disintegration, the amount of exposed DNA increased, thereby increasing the fluorescence intensity. This indicated that pore channels appeared first in the cytoplasmic membrane during the formation of NETs induced by PMA. Following this, cell membrane rupture occurs, resulting in a large number of NETs being released. The morphology of neutrophils treated with Nec-1 and the process of nuclear disaggregation and disintegration after PMA stimulation were consistent with those after PMA stimulation alone; however, the increase in the rate of green fluorescent-labelled DNA was slower and the range was smaller. The integrated density of the four groups suggested that the PMA group had significantly more NETs released than the PMA plus Nec-1 (100 *μ*M) group (*P* < 0.01). The significant reduction in the release of NETs could be observed at 120–240 min (Figure 2(d)).

These results suggested that PMA could induce DNA disintegration and release to form NETs. However, Nec-1 reduced PMA-induced cell membrane rupture and NET release in a dose-dependent manner. We speculate that neutrophils could release NETs through the pores in the cell membrane and Nec-1 could slow down this process.

### 2.3. Nec-1 Alleviated Lupus Pulmonary Haemorrhage in Mice

**Figure 3:**
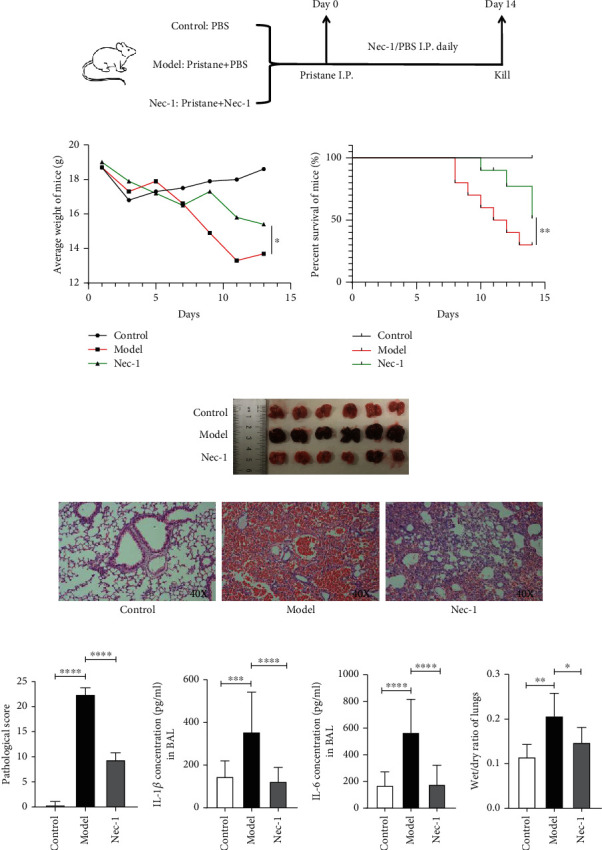
Nec-1 alleviated lupus pulmonary haemorrhage. (a) The modelling process of C57BL/6 mice. Model and Nec-1 group mice received the same dose of pristane/phosphate-buffered saline (PBS) via intraperitoneal injection (i.p.) on day 0, followed by daily i.p. injection of PBS/Nec-1 until sacrifice on day 14. (b) The weight-change trends of mice in different groups. (c) Survival curves of mice in different groups. (d) Morphology of lung tissues in different groups. (e) Representative images of the haematoxylin and eosin-stained lung tissues of mice from different groups. (f) Pathological staining score of mice lung tissue from different groups. (g, h) Levels of IL-1*β* and IL-6 in the bronchoalveolar lavage from different groups. (i) Wet/dry weight ratio of the mice lung tissue of different groups was calculated using ImageJ. Data are presented as mean ± standard deviation. Values are expressed as mean ± standard error of the mean of three independent experiments (^∗^*P* < 0.05, ^∗∗^*P* < 0.01, ^∗∗∗^*P* < 0.001, and ^∗∗∗∗^*P* < 0.0001).

We used a pristane-induced mouse model of DAH to verify whether Nec-1 could be used in the treatment of pulmonary haemorrhage in SLE (Figure 3(a)). We observed that the average weight of mice in the DAH model group was significantly lower than that of the control and Nec-1 groups (*P* < 0.05) (Figure 3(b)). Additionally, the survival rate of the model group was much lower than that of the control and Nec-1 groups (*P* < 0.01) (Figure 3(c)). The representative image showed that the lung tissue from the model group had an obvious increase in congestion while only a few parts of the lung tissue in the Nec-1 group had congestion. The overall degree of lung damage was lesser than that in the model group (Figure 3(d)). Histopathological studies on the stained paraffin sections of the lung tissues showed that the degree of inflammation and haemorrhage in the lung tissues of the Nec-1 group was lesser than that in the model group. In the model group, the lung alveoli appeared to be almost destroyed, the bronchial walls had collapsed, and cilia were absent (Figure 3(e)). The pathological staining score of the model group was also higher than that of the Nec-1 group (*P* < 0.0001) (Figure 3(f)). To further observe the degree of inflammation in DAH, we used inflammatory factor ELISA kits to quantify IL-1*β* and IL-6 levels in the bronchoalveolar lavage (BAL). The expression of IL1*β* and IL-6 in the BAL of the model group was significantly higher than that in the Nec-1 group (*P* < 0.0001) (Figures 3(g) and 3(h)). Furthermore, we determined the degree of lung oedema in each group based on the wet/dry weight ratio. The extent of oedema in the lung tissue of the model group, on average, was higher than that in the Nec-1 group (*P* < 0.05) (Figure 3(i)). These results thus indicate that Nec-1 could effectively reduce the inflammation and haemorrhage in the DAH model mice. We speculated that Nec-1 could be used as an inhibitor of NET production, thereby alleviating disease symptoms in the DAH model mice.

### 2.4. Nec-1 Alleviated Lupus Pulmonary Haemorrhage by Inhibiting the Release of NETs

**Figure 4:**
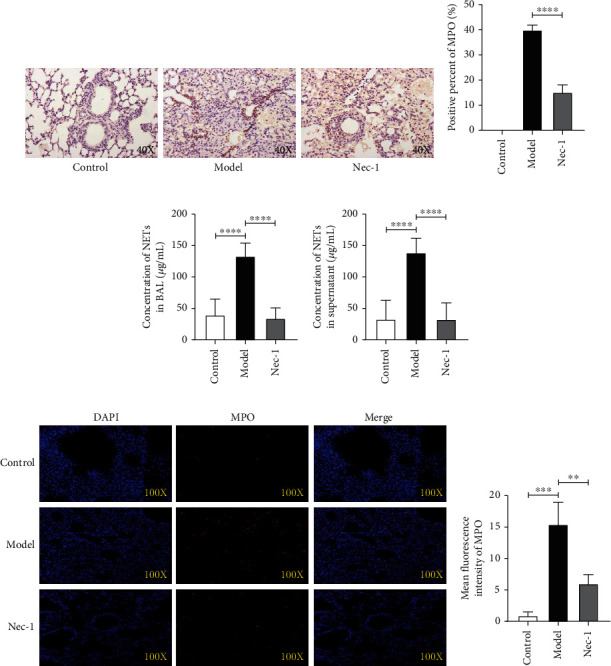
Nec-1 prevented the release of neutrophil extracellular traps (NETs) in mice with diffuse alveolar haemorrhage. (a) Representative images of the immunohistochemistry of MPO from different groups. (b) The positive rate of MPO expression in different groups was calculated using ImageJ. (c) The mean fluorescence intensity of SYTOX Green-stained extracellular DNA in the bronchoalveolar lavage (BAL) of mice was measured using a microplate reader. (d) The mean fluorescence intensity of SYTOX Green-stained extracellular DNA in the peripheral blood supernatant was measured using a microplate reader. (e) Representative immunofluorescence images of the nucleus and myeloperoxidase (MPO) in lung tissues from different groups. Nuclei were stained with 4′,6-diamidino-2-phenylindole (DAPI) (blue). MPO was stained with a fluorescent secondary antibody (red). The merge channel was mixed with two different colours. (f) The average fluorescence intensity of MPO in different groups was calculated using ImageJ. Data are presented as mean ± standard deviation. Values were expressed as mean ± standard error of the mean of three independent experiments (^∗∗^*P* < 0.01, ^∗∗∗^*P* < 0.001, and ^∗∗∗∗^*P* < 0.0001).

MPO is mainly expressed in neutrophils [4]. Immunohistochemical studies were used to detect MPO expression in the lung tissues, which revealed increased MPO expression in the lung tissue of the model group than in the Nec-1 group (Figure 4(a)). Positive rate statistics of immunohistochemical analysis suggested that MPO-positive cells in the model group were significantly higher than in the Nec-1 group (*P* < 0.0001) (Figure 4(b)). Then, we quantified NETs in the BAL and peripheral blood supernatant using SYTOX Green. The results showed that the expression of NETs in the BAL from the Nec-1 group was significantly lesser than that in the model group (*P* < 0.0001) (Figure 4(c)). Similar findings were observed in the peripheral blood supernatant (*P* < 0.0001) (Figure 4(d)). Then, immunofluorescence was used to stain the lung tissue. The captured images showed three channels of DAPI (blue), MPO (red), and merge (Figure 4(e)). The fluorescence intensity of MPO in the model group was more than that in other groups. ImageJ was used to perform the fluorescence quantification of the three groups, revealing the same results (*P* < 0.01) (Figure 4(f)). Furthermore, NET expression in the model group was significantly higher than those in the control group. This suggested that the formation of NETs could be inhibited by Nec-1 in mice.

### 2.5. Nec-1 Prevented the Release of NETs by Inhibiting the Activation of NE/GSDMD

**Figure 5:**
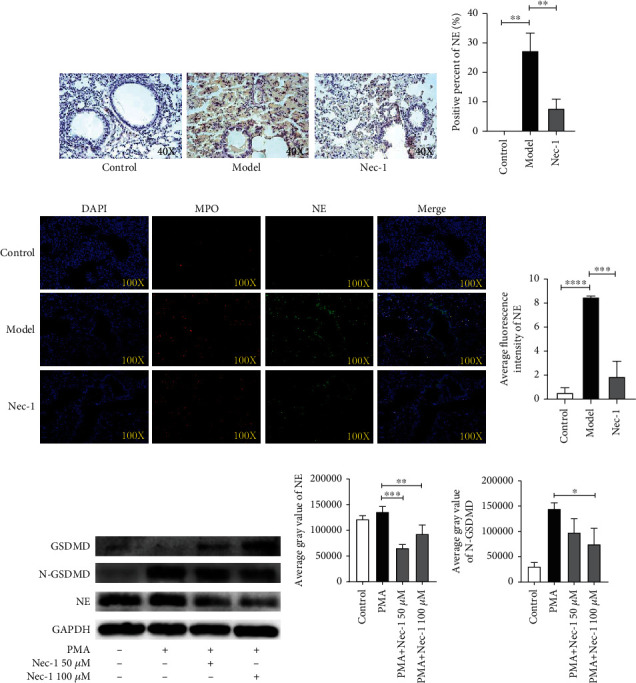
Nec-1 prevents the release of neutrophil extracellular traps (NETs) by inhibiting neutrophil elastase (NE). (a) Representative images of the immunohistochemistry of NE in different groups. The sepia areas indicate active NE cells. (b) NE-positive rate of immunohistochemistry in different groups was calculated using ImageJ. (c) Representative immunofluorescence images of the nucleus, NE, and MPO in the lung tissues of different groups. Nuclei were stained by DAPI (blue), whereas MPO and NE were stained with antibody immunofluorescence separately. (d) The average fluorescence intensity of NE in different groups was calculated using ImageJ. (e) Representative western blot images of Gasdermin D (GSDMD), N-Gasdermin D (N-GSDMD), NE, and glyceraldehyde-3-phosphate dehydrogenase (GAPDH) in different groups. (f) The average grey value of protein NE in different groups was calculated using ImageJ. (g) The average grey value of protein N-GSDMD in different groups was calculated using ImageJ. Data are presented as mean ± standard deviation. Values were expressed as mean ± standard error of the mean of three independent experiments (^∗^*P* < 0.05, ^∗∗^*P* < 0.01, ^∗∗∗^*P* < 0.001, and ^∗∗∗∗^*P* < 0.0001).

A previous study reported that Nec-1 inhibited PMA-stimulated NET formation; however, the exact mechanism was unclear [20]. NE, which can depolymerize chromatin and shred GSDMD to form N-GSDMD, and N-GSDMD eventually cause cell membrane rupture and NET release [22]. We conducted immunohistochemistry to detect NE expression in the lung tissue from each group (Figure 5(a)). The immunofluorescence histochemical assay revealed that the lung tissues from the Nec-1 group had fewer NE-positive cells compared with the model group (*P* < 0.01) (Figure 5(b)). Additionally, immunofluorescence staining for NE in lung tissues of different groups (Figure 5(c)) revealed that the level of NE in the model group was higher than that in the control and Nec-1 groups (*P* < 0.001) (Figure 5(d)). To verify whether there is a correlation between the expression of NE and N-GSDMD, western blot was performed, which revealed that the expressions of protein NE and N-GSDMD in normal human neutrophils were stimulated under different conditions (Figure 5(e)). The protein contents of NE and N-GSDMD in healthy human neutrophils stimulated by PMA alone were significantly higher than those in cells stimulated by PMA+plus Nec-1 (50 *μ*M and 100 *μ*M). The grey value quantitative analysis results of NE revealed similar results (*P* < 0.05) (Figure 5(f)). However, in the grey value analysis of N-GSDMD, only the PMA plus Nec-1 100 *μ*M group had a significant difference compared with the PMA group (*P* < 0.05) (Figure 5(g)). In the in vivo experiments, the levels of NE and MPO in the Nec-1 group were significantly decreased, indicating that the release of NETs was completely inhibited by Nec-1 in DAH mice. Thus, the findings from our in vitro experiments supported our hypothesis that Nec-1 inhibited NE and N-GSDMD activation to prevent the release of NETs.

## 3. Discussion

DAH is a serious complication that is responsible for the high mortality rate in patients with SLE. Previous studies have reported that patients with pulmonary haemorrhage in lupus have high neutrophil infiltration rates and NET formation in the airways [2, 3]. To mimic DAH in lupus, the C57BL/6 (B6) mouse model of pristane-induced lupus was developed, manifesting with alveolar and perivascular inflammation, capillaritis and small-vessel vasculitis, haemorrhage, and endothelial injury. Massive inflammatory cells, mainly neutrophils and macrophages, infiltrated the lungs of model mice [27, 28]. In 2019, Jarrot et al. first reported that neutrophils releasing NETs are an important cause of pulmonary haemorrhage in lupus [10]. Using antibodies for neutrophil depletion can reduce NET release and alleviate vascular inflammation in the lung tissue and pulmonary haemorrhage in the DAH mice model [27–29]. Nec-1 as an inhibitor of necrosis prevents cell necrosis through the RIPK1/RIPK3/MLKL pathway [30–32]. Previous studies have indicated that Nec-1 could effectively inhibit the formation of NETs stimulated by PMA. Furthermore, this study also reveals that NETs could directly damage human lung cancer cells in in vitro experiments. The C57BL/6 mice with lupus that were induced by pristane exhibited haemorrhage and pulmonary capillaritis, which is morphologically similar to that observed in human DAH caused by SLE. As expected, we found that Nec-1 effectively delayed the development of DAH. These findings validate the possibility that DAH can be treated by inhibiting the production of NETs in human lungs. However, further exploration is required to elucidate the specific mechanism.

**Figure 6:**
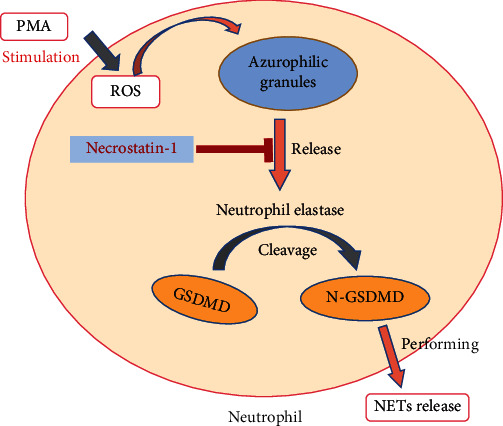
Schematic illustration of Necrostatin-1 (Nec-1) ameliorating diffuse alveolar haemorrhage in systemic lupus erythematosus by inhibiting the activation of neutrophil elastase (NE)/N-GSDMD to prevent neutrophil extracellular trap (NET) release.

Recent studies have shown that neutrophils release NETs and cell contents through pores in the cell membrane formed by N-GSDMD [33, 34]. NE could depolymerize chromatin and shred GSDMD to N-GSDMD, which forms pores in the cell membrane [35, 36]. To further study the mechanism by which Nec-1 inhibited the occurrence of DAH, the expressions of MPO and NE were detected in the lung tissue using immunohistochemistry and immunofluorescence. The expression of MPO and NE in the Nec-1 group was lower than that in the model group. Following this, human neutrophils were used to determine the effects of NE and N-GSDMD on human neutrophil membranes. The results showed that NE and N-GSDMD had destructive effects on the cell membrane, which could be attributed to the formation of pores in the cell membrane by N-GSDMD. Additionally, we hypothesise that Nec-1 could inhibit NE-cleaving GSDMD to form N-GSDMD, thereby preventing the release of NETs (Figure 6). Owing to the lack of antibodies of N-GSDMD for immunohistochemistry and immunofluorescence analyses, there is currently no evidence regarding the reduction in the expression of N-GSDMD in vivo.

In conclusion, this study identified that Nec-1, an inhibitor of necrosis, could alleviate lupus pulmonary haemorrhage by inhibiting the release of NETs. A possible mechanism is the inhibition of NE and N-GSDMD expressions by Nec-1. Thus, this study highlights the novel therapeutic implications of Nec-1 in the treatment of DAH.

## 4. Methods and Materials

### 4.1. In Vitro Experiments

#### 4.1.1. Neutrophil Isolation

We recruited healthy volunteers from The Third Affiliated Hospital of Southern Medical University. Venous peripheral blood was collected from each healthy volunteer. The entire process was performed according to the medical ethics guidelines. To obtain purified healthy human neutrophils, we used a neutrophil separation solution (Axis-Shield, Scotland, Cat No. AS1114684) for density-gradient separation. After separation, purified healthy human neutrophils were cultured with serum-free RPMI-1640 basal medium (Gibco, Thermo Fisher, USA, Cat No. 31870082) briefly until the subsequent step.

#### 4.1.2. Drugs

Phorbol 12-myristate 13-acetate (PMA) (Sigma-Aldrich, USA, Cat No.) at an original concentration of 1 mg/mL was diluted with phosphate-buffered saline (PBS, Thermo Fisher, USA, Cat No.10010023) to a working solution of 1 *μ*g/mL. Nec-1 (Merck, Germany, Cat No. 480065) at an original concentration of 5 mg/mL was diluted with PBS to a working solution of 1 mg/mL.

#### 4.1.3. PMA Stimulated Neutrophils to Produce NETs

The neutrophils obtained from the peripheral blood of the healthy volunteers were inoculated into 96-well plates (2 × 10^5^ cells/well). Different concentrations of Nec-1 were added for prestimulation. Then, the 96-well plates were incubated at 37°C and 5% carbon dioxide (CO_2_) for 30 min, followed by the addition of 20 nM PMA to each well to stimulate the production of NETs.

#### 4.1.4. Induction, Isolation, and Quantification of NETs

NETs were produced by PMA-stimulated neutrophils. Peripheral blood neutrophils (PBNs) were isolated from healthy donors, seeded onto 6-well plates (10^7^ cells/well), and stimulated by PMA at a concentration of 500 nM for 3 h. Supernatants were discarded. Neutrophils at the bottom of each dish were washed with 10 mL of cold PBS to remove the remaining appendiculate cells. The cells collected from each dish were centrifuged at 450 g and 4°C for 10 min. Neutrophils settled at the bottom of the tube, leaving a no-cell and NET-rich supernatant. The NET concentration was quantified using a Quant-iTTM PicoGreen dsDNA kit (Invitrogen, Paisley, UK, Cat No. P7589) [7, 15].

#### 4.1.5. Effect of NETs on the Viability of A549 Cells

A549 cells were donated by the Institute of Pathophysiology, College of Life Science and Technology, Southern Medical University. A549 cells were stored at 37°C in a 5% CO_2_ cell incubator for culturing. The appropriate amount of A549 cells was added to a 96-well plate (5^∗^10^4^ cells/well) and then treated with different concentrations of NETs. The 96-well plate was incubated at 37°C in a 5% CO_2_ environment for 3 h. The morphological changes of the cells were observed under an inverted light microscope (Olympus, Japan). A cell counting kit-8 (CCK-8) (Thermo Fisher, USA, Cat No. C7026) was used to detect cell proliferation. To each well, 10 *μ*L CCK8 solution was added, and the plate was incubated at 37°C in a 5% CO_2_ environment for 1 h. Absorbance (OD) at 450 nm was determined using a microplate reader (Molecular Devices, USA).

#### 4.1.6. Fluorescence Intensity Quantification of NETs

Neutrophils of healthy donors were stimulated using PMA (25 nM) for 2.5 h, and the impermeable DNA dye SYTOX Green (Thermo Fisher, USA, Cat No. S7020) was added to each well at a volume ratio of 1 : 100. Subsequently, fluorescence intensity was measured using a SpectraMax M5 multifunctional microplate reader (Biotek, USA) 10 min later. After PMA stimulation, the fluorescence intensity was recorded every 30 min for a total of 3.5 h.

#### 4.1.7. Observation of NETs Using Fluorescence Microscopy

Neutrophils of healthy donors were stimulated by PMA (25 nM) for 2.5 h, followed by the addition of the impermeable SYTOX Green DNA dye (Thermo Fisher, USA, Cat No. S7572) and the viable cell dye Hoechst 33342 (Beyotime, China, Cat No. C1025) at a volume ratio of 1 : 100 per well. NET production in each well was observed under an Olympus fluorescence microscope (Japan), and the randomly selected fields were photographed.

#### 4.1.8. Observation of Living Cells Using a Real-Time Imaging System

Neutrophils of healthy donors were stimulated by PMA (25 nM). The impermeable DNA dye SYTOX Green was added immediately at a volume ratio of 1 : 100 per well and analysed using a cell real-time imaging system (Biotek, USA). Bright-field (phase) and fluorescence (GFP) channels were used to take pictures continuously. Each group was photographed for 4 h.

#### 4.1.9. Western Blotting

Neutrophils isolated from healthy human peripheral blood were divided into four groups: control, PMA (25 nM), PMA+Nec-1 (50 *μ*M), and PMA+Nec-1 (100 *μ*M). The cells were cultured in a 6-well plate (1 × 10^7^ cells/well) and stimulated by PMA (25 nM) for 2.5 h. The supernatant was discarded. Then, the sinking cells were lysed with cold RIPA (Thermo Fisher, USA, Cat No. 89901) for 40 min. Protein samples were collected after centrifugation at 13000 rpm and 4°C for 5 min. A BCA kit (Thermo Fisher, USA, Cat No. 23225) was used to determine protein concentration. Protein samples were separated using sodium dodecyl-sulfate polyacrylamide gel electrophoresis (30 *μ*g per lane) and transferred to a polyvinylidene fluoride (PVDF) membrane (Merck, Germany, Cat No. ISEQ00005).

Membranes were blocked using 5% skim milk (Oxid, UK, Cat No. LP0031B) for 1 h, and NE (1 : 1000 dilution, Abcam, France, Cat No. ab68672), GSDMD (1 : 1000 dilution, Abcam, France, Cat No. ab210070), N-GSDMD (1 : 1000 dilution, Abcam, France, Cat No. ab215203), and glyceraldehyde-3-phosphate dehydrogenase (GAPDH) (1 : 1000 dilution, Origene, USA, Cat No. TA802519) antibodies were used for protein detection. The PVDF membranes treated with the antibodies were incubated overnight at 4°C and then washed thrice with PBS for 10 min each time. Membranes were further incubated with horseradish peroxidase-conjugated secondary antibodies (1 : 8000 dilution, Thermo Fisher, USA, Cat No. SA10001) for 1 h at room temperature and then washed thrice with PBS for 10 min each time. For the detection of target proteins, enhanced chemiluminescence (ECL) solution (Millipore, USA, Cat No. WBULS0100) was used on X-ray film. The exposure program was set to 5 s for GAPDH, 30 s for NE, and 1–3 min for GSDMD/N-GSDMD.

### 4.2. In Vivo Studies

#### 4.2.1. Animal Experiments

Female wild-type C57BL/6 mice aged 6–8 weeks and weighing 18 ± 2 g were obtained from the Guangdong Medical Laboratory Animal Center. Mice were raised in an SPF animal room. All animal experimental protocols were approved by the Animal Research Ethics Committee of The Third Affiliated Hospital of Southern Medical University.

#### 4.2.2. Establishment of the DAH Model

Mice were divided into three groups: control (*n* = 30), model (*n* = 30), and Nec-1 (*n* = 30). When all mice were up to at least 8 weeks of age, the modelling was initiated. Mice in the control group were daily administered 0.1 mL PBS (Thermo Fisher, USA, Cat No. 10010023) intraperitoneally. Mice in the model group were administered 0.8 mL pristane (Sigma-Aldrich, USA, Cat No. P2870) intraperitoneally only on the first day and subsequently administered 0.1 mL PBS intraperitoneally every day until their sacrifice [10, 16]. Mice in the Nec-1 group received an intraperitoneal injection of 0.8 mL pristane only on the first day. Then, 0.1 mL Nec-1 (ENZO, USA, Cat No. BML-AP309) at a dose of 6 mg/kg was administered daily until their sacrifice [15]. Nec-1 was diluted with PBS before administration. Every mouse's intraperitoneal injection of PBS and Nec-1 was completed within 1 min.

The body weights of mice were recorded every other day until the day before their sacrifice. All mice were anaesthetized, and their orbital blood was obtained and centrifuged at 4°C, 2000 rpm for 10 min to obtain the supernatant, which was stored at -80°C. Next, the thoracic cavities of the mice were surgically opened, and the lungs were carefully extracted and rinsed in precooled PBS. Mice lungs, which did not undergo operations, were lavaged thrice with precooled PBS (0.2 mL). The bronchoalveolar lavage (BAL) was centrifuged at 4°C at 2000 rpm for 5 min, and the supernatants were stored at -80°C. It should be noted that the internal structure of the lungs was destroyed after obtaining the BAL and hence could not be used for further experiments. The lung samples used for making sections were soaked in 4% paraformaldehyde or O.C.T. compound (Sakura, USA, Cat No.4583). Lungs soaked in O.C.T. compound were frozen at -20°C. The remaining lung samples were used to determine the wet/dry weight ratio.

#### 4.2.3. Wet/Dry Weight Ratio

The lungs were weighed using a weighing scale, and each lung was weighed thrice to obtain the average wet weight. After weighing, the lungs were combined and dried in an oven at 60°C for 48 h, after which, each lung was weighed and returned to the oven for further drying. This process was performed until the weight was constant, which was considered the final dry weight of the lungs. The dried lungs were weighed thrice, and the average dry weight of each lung was obtained. The weight of the lungs in each group was recorded.

#### 4.2.4. Quantification of NETs in the BAL and Peripheral Blood Supernatant

Quant-iTTM PicoGreen dsDNA kit (Invitrogen, Paisley, UK, Cat No. P7589) was used to quantify the DNA concentration in the BAL and peripheral blood supernatant following the manufacturer's instructions. A total of 100 *μ*L BAL or peripheral blood supernatant was added to each well. Then, 1 *μ*L PicoGreen solution was added to each well in a dark environment, and the plate was incubated at 37°C in a 5% CO_2_ environment for 20 min. Following this, the cells were excited at 480 nm and the fluorescence emission intensity was measured at 520 nm using a microplate reader. We calculated the concentration of NETs based on the standard curve.

#### 4.2.5. Quantification of IL-1*β* and IL-6 in the BAL

The manufacturer's instructions in the ELISA test kit were followed to determine IL-1*β* (Neobioscience, China, Cat No. EMC001b.96) and IL-6 (Neobioscience, China, Cat No. EMC004.96) concentrations in the BAL. A standard curve was drawn at 0-1000 pg/mL concentration. A total of 100 *μ*L BAL of the three groups was added to each well of a 96-well plate and incubated at 37°C in a 5% CO_2_ environment for 90 min. Subsequently, a 100 *μ*L biotinylated antibody was added to each well, and the plate was incubated at 37°C in a 5% CO_2_ environment for 60 min. An enzyme conjugate (100 *μ*L) was added to each well plate and incubated at 37°C in a 5% CO_2_ environment for 30 min. Before adding the next liquid, the plate was washed five times, discarding the wash liquid from the plate at every wash. Then, 100 *μ*L chromogenic substrate was added to each well, and the plate was incubated at 37°C in a 5% CO_2_ environment for 15 min. Finally, a reaction stop solution (100 *μ*L) was added to each well, and the absorbance at 450 nm was determined using a microplate reader. We calculated the concentration of IL-6 and IL-1*β* based on the standard curve.

#### 4.2.6. Paraffin Sections

The lungs were soaked in 4% paraformaldehyde for 24 h and embedded in paraffin, and 4 *μ*m thick paraffin sections were prepared using a semiautomatic microtome (Thermo Fisher, USA).

#### 4.2.7. Frozen Sections

The lungs were embedded with O.C.T. compound. A microtome cryostat (Leica, Germany) was used to obtain 10 *μ*M thick sections, which were frozen and stored at -20°C.

#### 4.2.8. Pathological Staining and Immunohistochemistry

A haematoxylin and eosin staining kit (Leagene, China, Cat No. DH0006-100) was used to stain the paraffin sections. Images were captured using an Olympus microscope.

Citric acid was used to repair the antigen of the paraffin sections overnight at 60°C. The sections were blocked with 1% hydrogen peroxide for 20 min at room temperature and then blocked with 4% goat serum (Sigma-Aldrich, USA, Cat No. I5256) for 40 min at room temperature, followed by overnight incubation with the MPO antibody (1 : 100 dilution, Abcam, France, Cat No. ab208670) and NE antibody (1 : 100 dilution, Abcam, France, Cat No. ab68672) at 4°C. Then, the sections were incubated with HRP-conjugated secondary antibody (1 : 200 dilution, Thermo Fisher, USA, Cat No. A16096) for 2 h at room temperature. Finally, a DAB chromogenic kit (Leagene, China, Cat No. PW0072) was used to visualise the positive expression of the proteins. Images were captured using an Olympus microscope.

#### 4.2.9. Immunofluorescence

Frozen sections were used for immunofluorescence analyses. The lung sections were blocked with 4% goat serum (Sigma-Aldrich, USA, Cat No. I5256) at room temperature for 40 min and incubated overnight with MPO antibody (1 : 100 dilution, R&D, USA, Cat No.AF3667) and NE antibody (1 : 100 dilution, Abcam, France, Cat No. ab21595) at 4°C. Next, the sections were incubated with secondary antibodies conjugated with Alexa Fluor 488 (1 : 200 dilution, Abcam, France, Cat No. ab150073) or Alexa Fluor 647 (1 : 200 dilution, Thermo Fisher, USA, Cat No. A-21447) at 4°C for 2 h. DAPI (Sigma-Aldrich, USA, D9542) was used to mount the sections and stain the nuclei. Images were captured using a fluorescent confocal microscope.

## 5. Statistical Analysis

The results are presented as mean ± standard error of the mean. Data were analysed using a one-tailed Student's *t*-test or one-way analysis of variance, followed by Tukey's post hoc test or the nonparametric Wilcoxon rank-sum test. All statistical analyses were performed using Prism7 (GraphPad Software, La Jolla, CA, USA). *P* < 0.05 was considered statistically significant. Every experiment was repeated at least thrice.

## Data Availability

All data generated or analysed in this study are included in this published article and its supplementary information files.

## Supplementary Materials

Supplementary Materials Supplementary videos: the change process of cells from 0 to 240 minutes in different groups pictured by the real-time imaging system.
