# Supplementary figures and images for: Necrostatin-1 Alleviates Diffuse Pulmonary Haemorrhage by Preventing the Release of NETs via Inhibiting NE/GSDMD Activation in Murine Lupus
Source: J Immunol Res. 2023 Mar 1;2023:4743975. doi: 10.1155/2023/4743975 (PMC9995194; doi:10.1155/2023/4743975)

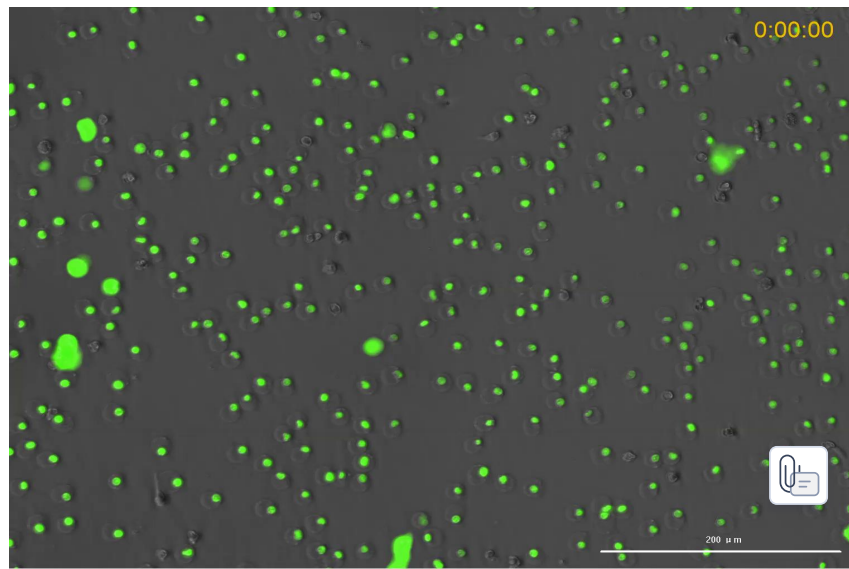

Control

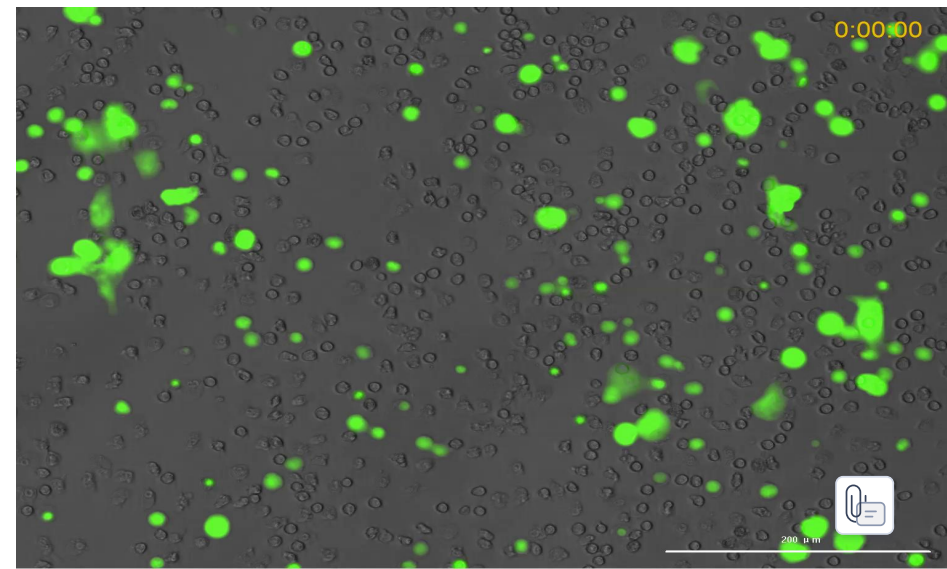

PMA

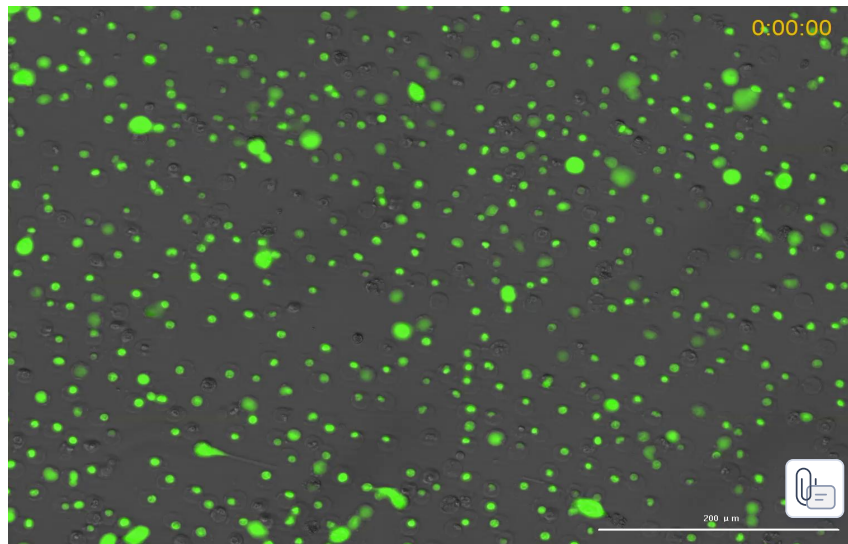

PMA+Nec-1 50  $\mu$ mol

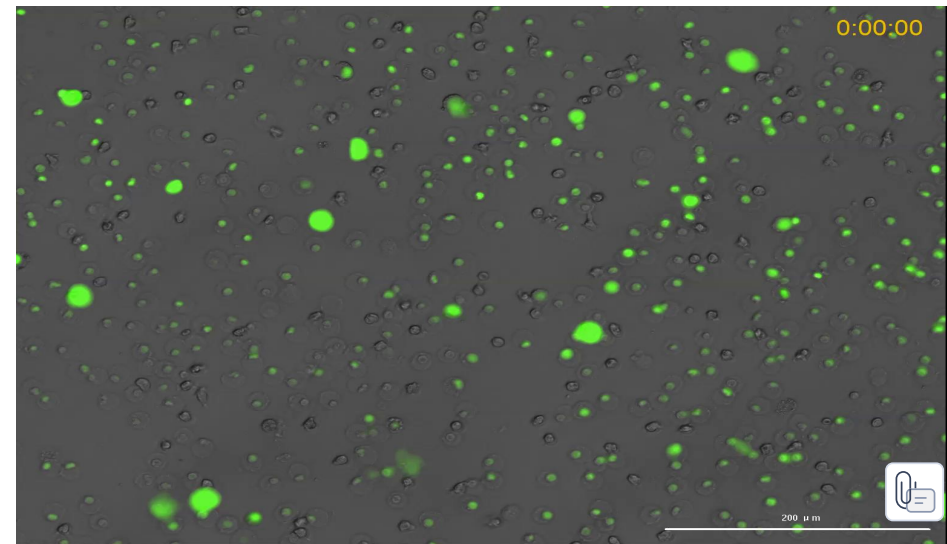

PMA+Nec-1 100  $\mu$ mol

Supplement: Supplementary Materials — Supplementary videos: the change process of cells from 0 to 240 minutes in different groups pictured by the real-time imaging system. [file 4743975.f1.pdf]
